# A Fractional Single-Phase-Lag Model of Heat Conduction for Describing Propagation of the Maximum Temperature in a Finite Medium

**DOI:** 10.3390/e20110876

**Published:** 2018-11-15

**Authors:** Stanisław Kukla, Urszula Siedlecka

**Affiliations:** Institute of Mathematics, Czestochowa University of Technology, 21 Armii Krajowej Ave., 42-201 Częstochowa, Poland

**Keywords:** fractional heat conduction, single-phase-lag model, propagation of the maximum temperature, Caputo derivative, Robotnov function

## Abstract

In this paper, an investigation of the maximum temperature propagation in a finite medium is presented. The heat conduction in the medium was modelled by using a single-phase-lag equation with fractional Caputo derivatives. The formulation and solution of the problem concern the heat conduction in a slab, a hollow cylinder, and a hollow sphere, which are subjected to a heat source represented by the Robotnov function and a harmonically varying ambient temperature. The problem with time-dependent Robin and homogenous Neumann boundary conditions has been solved by using an eigenfunction expansion method and the Laplace transform technique. The solution of the heat conduction problem was used for determination of the maximum temperature trajectories. The trajectories and propagation speeds of the temperature maxima in the medium depend on the order of fractional derivatives occurring in the heat conduction model. These dependencies for the heat conduction in the hollow cylinder have been numerically investigated.

## 1. Introduction

The classical Fourier’s law of the heat conduction establishes the relationship between the heat flux vector and the gradient of the temperature [1]

(1) q(r,t)=−k∇T(r,t)

where **q** is the heat flux vector, **r** is the point in the considered region, *t* is the time, *k* is the thermal conductivity of the material, ∇ is the gradient operator and *T* is the temperature. This relationship implies a nonphysical infinite speed of a thermal signal in the medium. To avoid this disagreement between the mathematical model and the observations, the single-phase-lag was introduced to the heat conduction model. Namely, the relationship (Equation (1)) is replaced by the following one [2]

(2) q(r,t+τ)=−k∇T(r,t)

where *τ* is the phase lag of the heat flux. Expanding the left-hand side of Equation (2) into the Taylor series with respect to variable *τ* and taking into account two terms of this series (assuming that the phase lag *τ* is small), Equation (2) can be approximated by

(3) $$q\left(r,t\right)+\tau \frac{\partial q}{\partial t}\left(r,t\right)=-k\nabla T\left(r,t\right)$$

Equation (3) was proposed by Cattaneo [3] and Vernotte [4] and currently it is known as the Cattaneo–Vernotte constitutive equation [5].

The heat flux vector occurring in Equation (3) can be eliminated by using the energy equation [6]

(4) $$-\nabla \cdot q\left(r,t\right)+g\left(r,t\right)=\rho C_{p}\frac{\partial T}{\partial t}$$

where g(r,t) is the rate of the heat generation per unit volume, ρ is the density of the material and $C_{p}$ is the specific heat capacity. As a result, the single-phase-lag heat conduction equation is obtained:

(5) $$\frac{\partial^{2}T}{\partial t^{2}}\left(r,t\right)+\frac{1}{\tau }\frac{\partial T}{\partial t}\left(r,t\right)=\frac{\kappa }{\tau }\nabla^{2}T\left(r,t\right)+\frac{\kappa }{k}\left(\frac{\partial g\left(r,t\right)}{\partial t}+\frac{1}{\tau }g\left(r,t\right)\right)$$

where $\kappa =k/\left(\rho C_{p}\right)$ is the thermal diffusivity. This hyperbolic type of equation describes the heat transfer in the wave form. The finite speed of the heat wave in the medium determines the square root of the ratio κ/τ. In the literature, there are numerous applications of the Equation (5) for modelling of the heat conduction. For instance, recently, works [7,8,9,10,11] have been published in which the hyperbolic model of heat conduction was applied.

The description of transport processes with the use of fractional derivatives was proposed by Compte and Metzler [12]. In the presented mathematical model, a generalization of the Cattaneo–Vernotte constitutive equation was utilized. This generalization consists of replacing the time-derivative in the constitutive equation (Equation (3)) by the fractional derivative. The resulting generalized constitutive equation has the following form:

(6) $$q\left(r,t\right)+\tau^{\alpha }\frac{\partial^{\alpha }q}{\partial t^{\alpha }}=-k\nabla T\left(r,t\right),\;0<\alpha \leq 1$$

where $\frac{\partial^{\alpha }}{\partial t^{\alpha }}$ denotes the fractional derivative with respect to variable *t* of order *α*. Scalar multiplying Equation (6) by the vector ∇ and then using Equation (4), the heat flux vector **q** can be eliminated. As a result, the fractional heat conduction equation is obtained:

(7) $$\frac{\partial^{\alpha }}{\partial t^{\alpha }}\left(\frac{\partial T}{\partial t}\left(r,t\right)\right)+\frac{1}{\tau^{\alpha }}\frac{\partial T}{\partial t}\left(r,t\right)=\frac{\kappa }{\tau^{\alpha }}\nabla^{2}T\left(r,t\right)+\frac{\kappa }{k}\left(\frac{\partial^{\alpha }g\left(r,t\right)}{\partial t^{\alpha }}+\frac{1}{\tau^{\alpha }}g\left(r,t\right)\right)$$

A large variety of generalizations of the constitutive equation for the heat transfer and their applications are presented in the literature [13,14,15,16,17,18,19]. 

A generalization of the heat conduction model can be obtained by replacing the time derivative in Equation (4) with the fractional derivative of order *β*. The obtained generalized fractional energy equation has the form

(8) $$-\nabla \cdot q\left(r,t\right)+g\left(r,t\right)=\rho C_{p}\nu^{\beta -1}\frac{\partial^{\beta }T}{\partial t^{\beta }},\;0<\beta \leq 1$$

In this equation, the coefficient $\nu^{\beta -1}$ is introduced to keep the accordance of dimensions. The generalized constitutive equation (Equation (6)) in combination with the generalized energy equation (Equation (8)), results in the single-phase-lag heat conduction equation with fractional derivatives

(9) $$\frac{\partial^{\alpha }}{\partial t^{\alpha }}\left(\frac{\partial^{\beta }T}{\partial t^{\beta }}\left(r,t\right)\right)+\frac{1}{\tau^{\alpha }}\frac{\partial^{\beta }T}{\partial t^{\beta }}\left(r,t\right)=\frac{\kappa }{\tau^{\alpha }\nu^{\beta -1}}\nabla^{2}T\left(r,t\right)+\frac{\kappa }{k\nu^{\beta -1}}\left(\frac{\partial^{\alpha }g\left(r,t\right)}{\partial t^{\alpha }}+\frac{1}{\tau^{\alpha }}g\left(r,t\right)\right)$$

The notation of the first term on the left-hand side of Equation (9) is dictated by the fact that in general, the fractional derivatives are noncommutative operators [20]. It can be noted that this equation for β=1 is of the form of fractional Equation (7), and for α=β=1, it has the form of hyperbolic Equation (5). In the literature, there are no works devoted to the propagation of the maximum temperature in a medium based on the heat conduction model in which the non-local and phase-lag properties are considered.

In applications of the fractional calculus, the Riemann–Liouville and the Caputo derivatives are often used. The definitions and properties of these fractional derivatives are presented in the books by Diethelm [21], Kilbas et al. [22], Mainardi [20], Podlubny [23] and Povstenko [24]. In this paper, the Caputo derivative and its properties will be used. The fractional Caputo derivative of order *α* with the lower limit zero, is defined as

(10) ∂αf(r,t)∂tα=Dtα0Cf(r,t)={1Γ(n−α)∫0t(t−τ)n−α−1∂nf(r,τ)∂τndτ, n−1<α<n∂nf(r,t)∂tn, α=n, n∈N

From Equation (10), it follows that the fractional derivatives occurring in the differential equation describing the state of the system contain information about its past state. This non-local property of the fractional derivatives is the important advantage of using fractional calculus in mathematical modeling.

The fractional differential Equation (9) is completed by initial and boundary conditions. A solution to this initial–boundary value problem is the temperature distribution as a function of time and space variables. This function for a fixed time variable can achieve a local maximum value with respect to the space variable. The point of the maximum propagates with a finite speed in the considered region. The propagation problem of the maximum point of a fundamental solution to a fractional equation was considered by Luchko et al. [25]. The presented results concern the Cauchy problem for a one-dimensional time-fractional diffusion-wave equation in an unbounded region.

In this paper, a solution to the heat conduction problem according to the time-fractional single-phase-lag model is presented. The solution in the Laplace transform domain includes the one-dimensional heat conduction in a slab, a hollow cylinder, and a hollow sphere. The obtained temperature distribution for the tracking of the propagation of the maximum temperature in the considered region was used. The presented numerical results concern the hollow cylinder with the Robin–Neumann boundary conditions, which is subjected to a variable ambient temperature or impulsive heat source.

## 2. Formulation of the Problem

Let us consider the heat conduction governed by the time-fractional differential equation (Equation (9)). This equation is valid in the region which is specified by a medium in the space. We will deal with heat conduction in a slab, a hollow cylinder, and a hollow sphere. In the each of the three cases, assuming one dimensional heat conduction, we denote the space variable by “*x*” where a≤x≤b. For the slab, the heat conduction in the direction of *x*-axis of a rectangular coordinate system is considered, for the cylinder—in a radial direction of a cylindrical coordinate system and for the sphere—in a radial direction of a spherical coordinate system. The operator $\nabla^{2}$ in Equation (9) for the slab, cylinder and sphere can be written in the form [1]

(11) $$\nabla^{2}T=\frac{1}{x^{p}}\frac{\partial }{\partial x}\left(x^{p}\frac{\partial T}{\partial x}\right),\;p=0,1,2$$

where p=0 for the slab, p=1 for the cylinder and p=2 for the sphere.

Equation (9) is complemented by boundary and initial conditions. We assume the Robin–Neumann boundary conditions:

(12) $$k\frac{\partial T}{\partial x}\left(a,t\right)=-h_{a}\left(T_{a}\left(t\right)-T\left(a,t\right)\right)$$

(13) $$\frac{\partial T}{\partial x}\left(b,t\right)=0$$

and the following initial conditions:

(14) T(x,0)=f(x)

(15) $$\frac{\partial^{\beta }T}{\partial t^{\beta }}\left(x,0\right)=h\left(x\right)$$

where $h_{a}$ is the convective heat transfer coefficient, $T_{a}\left(t\right)$ is the ambient temperature, f(x) is the initial temperature, and h(x) is the fractional time-derivative of order *β* of the temperature at an initial time. 

The function T(x,t) is a solution of the initial–boundary problem (Equations (9) and (12)–(15)) with a non-homogenous boundary condition. In the first stage of solving this problem, we present the function T(x,t) in the form of the sum

(16) $$T\left(x,t\right)=T_{a}\left(t\right)+\theta \left(x,t\right)$$

The function θ(x,t) satisfies the non-homogenous differential equation

(17) $$\frac{\partial^{\alpha }}{\partial t^{\alpha }}\left(\frac{\partial^{\beta }\theta }{\partial t^{\beta }}\right)+\frac{1}{\tau^{\alpha }}\frac{\partial^{\beta }\theta }{\partial t^{\beta }}=\frac{\kappa }{\tau^{\alpha }\nu^{\beta -1}}\nabla^{2}\theta +G\left(x,t\right)-Q\left(t\right)$$

and the following homogenous boundary conditions

(18) $$k\frac{\partial \theta }{\partial x}\left(a,t\right)-h_{a}\theta \left(a,t\right)=0$$

(19) $$\frac{\partial \theta }{\partial x}\left(b,t\right)=0$$

where

(20a) $$G\left(x,t\right)=\frac{\kappa }{k\nu^{\beta -1}}\left(\frac{\partial^{\alpha }g\left(x,t\right)}{\partial t^{\alpha }}+\frac{1}{\tau^{\alpha }}g\left(x,t\right)\right)$$

(20b) $$Q\left(t\right)=\frac{d^{\alpha }}{dt^{\alpha }}\left(\frac{d^{\beta }T_{a}\left(t\right)}{dt^{\beta }}\right)+\frac{1}{\tau^{\alpha }}\frac{d^{\beta }T_{a}\left(t\right)}{dt^{\beta }}$$

The initial conditions for the function θ(x,t) are obtained using Equations (14)–(16)

(21) $$\theta \left(x,0\right)=f\left(x\right)-T_{a}\left(0\right)$$

(22) $$\frac{\partial^{\beta }\theta }{\partial t^{\beta }}\left(x,0\right)=h\left(x\right)-{\frac{d^{\beta }T_{a}}{dt^{\beta }}|}_{t=0}$$

The function θ(x,t) as a solution of the initial–boundary problem (Equations (17)–(22)), will be determined in the form of an orthogonal series, and then the Laplace transform technique will be used.

## 3. Solution to the Problem

We can search for a solution to the problem (Equations (17)–(22)) in the form of a series:

(23) $$\theta \left(x,t\right)=\sum_{i=1}^{\infty }\Lambda_{i}\left(t\right)\Phi_{i}\left(x\right)$$

where the functions $\Phi_{i}\left(x\right)$ are solutions of the following eigenproblem

(24) $$\nabla^{2}\Phi \left(x\right)+\lambda^{2}\Phi \left(x\right)=0$$

(25) $$k\Phi^{\prime }\left(a\right)-h_{a}\Phi \left(a\right)=0$$

(26) $$\Phi^{\prime }\left(b\right)=0$$

The general solution to Equation (24) can be written in the form

(27) Φ(x)=Aφ(x)+Bψ(x)

where *A*, *B* are constants and the functions φ(x) and ψ(x) are independent, particular solutions to this equation. Taking into account the boundary conditions (Equations (25) and (26)) and using the standard procedure, we obtain the eigenvalue equation

(28) $$\left(k\varphi^{\prime }\left(a\right)-h_{a}\varphi \left(a\right)\right)\psi^{\prime }\left(b\right)-\left(k\psi^{\prime }\left(a\right)-h_{a}\psi \left(a\right)\right)\varphi^{\prime }\left(b\right)=0$$

which is solved with respect to λ. The obtained roots create an infinite sequence of eigenvalues: $\lambda_{i}$, i=1,2,…. In turn, assuming $A_{i}=\psi_{i}^{\prime }\left(b\right)$ and using Equations (26) and (27) for $\lambda =\lambda_{i}$, i=1,2,…, we find $B_{i}=-\varphi_{i}^{\prime }\left(b\right)$. Hence, the eigenfunctions $\Phi_{i}\left(x\right)$ corresponding to the eigenvalues $\lambda_{i}$ can be rewritten as

(29) $$\Phi_{i}\left(x\right)=\psi_{i}^{\prime }\left(b\right)\varphi_{i}\left(x\right)-\varphi_{i}^{\prime }\left(b\right)\psi_{i}\left(x\right),\;\mathrm{for}\;i=1,2,\ldots$$

The functions $\varphi_{i}\left(x\right)$ and $\psi_{i}\left(x\right)$ for the three cases of the operator $\nabla^{2}$ given by Equation (11) are presented in Table 1.

The functions $\Phi_{i}\left(x\right)$ satisfy the orthogonality condition in the form

(30) $$\int_{a}^{b}x^{p}\Phi_{i}\left(x\right)\Phi_{j}\left(x\right)dx=\{\begin{array}{cc} 0 & \mathrm{for}\;i\neq j\\ N_{i} & \mathrm{for}\;i=j \end{array}$$

where the normalization integrals $N_{i}$ are determined according to the formula

(31) $$N_{i}=\int_{a}^{b}x^{p}{\left(\Phi_{i}\left(x\right)\right)}^{2}dx$$

The eigenfunctions $\Phi_{i}\left(x\right)$, eigenvalue equations, and normalization integrals $N_{i}$ for the eigenvalue problem (Equations (24)–(26)) for a slab, a hollow cylinder, and a hollow sphere, are presented in Table 2. The presented approach can be applied to the fractional single-phase-lag heat conduction problem obtained by replacing the Neumann boundary condition (Equation (13)) with the homogeneous Dirichlet boundary condition: T(b,t)=0.

In order to derive an equation that will be used to determine the functions $\Lambda_{i}\left(t\right)$ occurring in Equation (23), we substitute the series (Equation (23)) into Equation (17), then we multiply the resulting equation by the function $x^{p}\Phi_{j}\left(x\right)$ and we integrate it over the interval [a,b]. Using the condition from Equation (30), we obtain the equation in the form

(32) $$\frac{d^{\alpha }}{dt^{\alpha }}\left(\frac{d^{\beta }\Lambda_{i}}{dt^{\beta }}\right)+\frac{1}{\tau^{\alpha }}\frac{d^{\beta }\Lambda_{i}}{dt^{\beta }}+\frac{\kappa }{\tau^{\alpha }\nu^{\beta -1}}\lambda_{i}^{2}\Lambda_{i}=\frac{1}{N_{i}}\int_{a}^{b}x^{p}\Phi_{i}\left(x\right)G\left(x,t\right)dx-\frac{Q\left(t\right)}{N_{i}}\int_{a}^{b}x^{p}\Phi_{i}\left(x\right)dx$$

Similarly, multiplying both sides of Equations (21) and (22) by $x^{p}\Phi_{j}\left(x\right)$ and integrating over the interval [a,b], the following initial conditions are obtained:

(33) $$\Lambda_{i}\left(0\right)=\frac{1}{N_{i}}\int_{a}^{b}x^{p}\Phi_{i}\left(x\right)\left(f\left(x\right)-T_{a}\left(0\right)\right)dx$$

(34) $$\frac{d^{\beta }\Lambda_{i}}{dt^{\beta }}\left(0\right)=\frac{1}{N_{i}}\int_{a}^{b}x^{p}\Phi_{i}\left(x\right)\left(h\left(x\right)-{\frac{d^{\beta }T_{a}}{dt^{\beta }}|}_{t=0}\right)dx$$

We find a solution to the initial problem (Equations (32)–(34)) by using the Laplace transform technique. The Laplace transform $L\left[f\left(t\right)\right]=\bar{f}\left(s\right)$ of a function f(t) is defined as

(35) $$\bar{f}\left(s\right)=\int_{0}^{\infty }f\left(t\right)e^{-st}dt$$

where s is a complex parameter. We utilized the linearity property of the Laplace transform and the following rule [20]:

(36) $$L\left\{\frac{d^{\mu }}{dt^{\mu }}f\left(t\right)\right\}=s^{\mu }F\left(s\right)-\sum_{k=0}^{m-1}s^{\mu -1-k}f^{\left(k\right)}\left(0^{+}\right),\;m-1<\mu \leq m$$

Using the rule (Equation (36)), the Laplace transform of the solution to the problem (Equations (32)–(34)), after some transformation, can be presented in the form

(37) $$\begin{array}{l} {\bar{\Lambda }}_{i}\left(s\right)=\frac{1}{N_{i}\sigma_{i}^{\alpha ,\beta }\left(s\right)}\left[\int_{a}^{b}x^{p}\Phi_{i}\left(x\right)\bar{G}\left(x,s\right)dx-\bar{Q}\left(s\right)\int_{a}^{b}x^{p}\Phi_{i}\left(x\right)dx\right]\\ +\frac{1}{N_{i}\sigma_{i}^{\alpha ,\beta }\left(s\right)}\left[\left(s^{\alpha +\beta -1}+\frac{1}{\tau^{\alpha }}s^{\beta -1}\right)\int_{a}^{b}x^{p}\Phi_{i}\left(x\right)\left(f\left(x\right)-T_{a}\left(0\right)\right)dx+s^{\alpha -1}\int_{a}^{b}x^{p}\Phi_{i}\left(x\right)\left(h\left(x\right)-{\frac{d^{\beta }T_{a}}{dt^{\beta }}|}_{t=0}\right)dx\right] \end{array}$$

where

(38) $$\sigma_{i}^{\alpha ,\beta }\left(s\right)=s^{\alpha +\beta }+\tau^{-\alpha }s^{\beta }+\tau^{-\alpha }\nu^{1-\beta }\kappa \lambda_{i}^{2}$$

and

(39) $$\bar{Q}\left(s\right)=\left(s^{\alpha }+\tau^{-\alpha }\right)\left(s^{\beta }{\bar{T}}_{a}\left(s\right)-s^{\beta -1}T_{a}\left(0\right)\right)-s^{\alpha -1}{\frac{d^{\beta }T_{a}\left(t\right)}{dt^{\beta }}|}_{t=0}$$

is the Laplace transform of the function Q(t) given by Equation (20b). The complete solution to the problem in the Laplace transform domain will be determined for an established function describing the rate of the heat generation g(x,t) and functions occurring in the initial and boundary conditions: f(x), h(x) and $T_{a}\left(t\right)$.

We find the Laplace transform $\bar{G}\left(x,s\right)$ assuming the function g(x,t) in Equation (20a) in the form

(40) $$g\left(x,t\right)=\frac{g_{0}}{2\pi a\tau^{\alpha }}F_{\alpha }\left(-\tau^{-\alpha },t\right)\delta \left(x-a\right)$$

where $g_{0}$ is the strength of the heat source per unit length of the surface [26], δ(·) is the Dirac delta function, $F_{\alpha }\left(\lambda ,z\right)$ is the Robotnov function [27]:

(41) $$F_{\alpha }\left(\lambda ,z\right)=z^{\alpha -1}E_{\alpha ,\alpha }\left(\lambda z^{\alpha }\right)$$

and $E_{\alpha ,\beta }\left(z\right)$ is the two-parameter Mittag–Leffler function defined by the power series

(42) $$E_{\alpha ,\beta }\left(z\right)=\sum_{n=0}^{\infty }\frac{z^{n}}{\Gamma \left(\alpha n+\beta \right)},\;\alpha >0,\;\beta >0$$

The Robotnov function is called the “impulse response” of the fundamental fractional order differential equation because it satisfies the differential equation [28]

(43) $$\frac{\partial^{\alpha }F_{\alpha }\left(-\lambda ,t\right)}{\partial t^{\alpha }}+\lambda F_{\alpha }\left(-\lambda ,t\right)=\delta \left(t\right)$$

Using Equations (38), (39) and (43) and the Laplace transform pair [24]

(44) $$L\left[t^{\beta -1}E_{\alpha ,\beta }\left(-\lambda t^{\alpha }\right)\right]=\frac{s^{\alpha -\beta }}{s^{\alpha }+\lambda }$$

the Laplace transform of the function G(x,t) defined by Equation (20a), can be written as

(45) $$\bar{G}\left(x,s\right)=\frac{g_{0}\kappa }{2\pi ak\tau^{\alpha }\nu^{\beta -1}}\delta \left(x-a\right)$$

The functions $T_{a}\left(t\right)$, f(x) and h(x) occurring in the boundary and initial conditions (12) and in Equations (14) and (15), we assume that

(46a) $$T_{a}\left(t\right)=P_{1}+P_{2}\sin\omega t,\;t\geq 0$$

(46b) $$f\left(x\right)=T_{a}\left(0\right),\;h\left(x\right)=0\;\mathrm{for}\;x\in \left[a,b\right]$$

Considering Equations (39) and (45), in Equation (37) we obtain the Laplace transform ${\bar{\Lambda }}_{i}\left(s\right)$ in the form

(47) $${\bar{\Lambda }}_{i}\left(s\right)=\frac{1}{N_{i}\sigma_{i}^{\alpha ,\beta }\left(s\right)}\frac{g_{0}\kappa }{2\pi ak\tau^{\alpha }\nu^{\beta -1}}a^{p}\Phi_{i}\left(a\right)-\frac{\left(s^{\alpha }+\tau^{-\alpha }\right)s^{\beta }}{N_{i}\sigma_{i}^{\alpha ,\beta }\left(s\right)}{\bar{T}}_{a}\left(s\right)\int_{a}^{b}x^{p}\Phi_{i}\left(x\right)dx$$

where

(48) $${\bar{T}}_{a}\left(s\right)=\frac{P_{1}}{s}+\frac{P_{2}\omega }{s^{2}+\omega^{2}}$$

For the purpose of deriving the inverse Laplace transform $L^{-1}\left[{\bar{\Lambda }}_{i}\left(s\right)\right]$, we use the convolution theorem [20]

(49) $$L^{-1}\left[\bar{f}\left(s\right)\bar{g}\left(s\right)\right]=\int_{0}^{t}f\left(u\right)g\left(t-u\right)du$$

Introducing the function $U_{i}^{\alpha ,\beta ,\gamma }\left(t\right)$ defined as

(50) $$U_{i}^{\alpha ,\beta ,\gamma }\left(t\right)=L^{-1}\left[\frac{s^{\gamma }}{\sigma_{i}^{\alpha ,\beta }\left(s\right)}\right]$$

and using the properties in Equation (49), we can write the inverse Laplace transform $\Lambda_{i}\left(t\right)$ in the form

(50) $$\begin{array}{ll} \Lambda_{i}\left(t\right)= & \frac{g_{0}\kappa }{2\pi ak\tau^{\alpha }\nu^{\beta -1}N_{i}}a^{p}\Phi_{i}\left(a\right)U_{i}^{\alpha ,\beta ,0}\left(t\right)\\ & -\frac{P_{2}\omega }{N_{i}}\int_{0}^{t}\left(\tau^{-\alpha }U_{i}^{\alpha ,\beta ,\beta -1}\left(u\right)+U_{i}^{\alpha ,\beta ,\alpha +\beta -1}\left(u\right)\right)\cos\omega \left(t-u\right)du\cdot \int_{a}^{b}x^{p}\Phi_{i}\left(x\right)dx \end{array}$$

Finally, the temperature distribution T(x,t) is given by Equations (16), (23), (27) and (51).

The function $U_{i}^{\alpha ,\beta ,\gamma }\left(t\right)$, as an inverse Laplace transform, can be determined in an analytical form but only for some values of the fractional orders α and β. For example, if α=0.5,β=1.0 and γ=0;0.5, this function can be written in the form

(52) $$U_{i}^{\alpha ,\beta ,\gamma }\left(t\right)=\begin{array}{cc} \sum_{j=1}^{3}\frac{Z_{ij}^{2\gamma }}{\zeta_{ij}}F_{0.5}\left(Z_{ij},t\right) & \mathrm{for}\;\gamma =0;0.5 \end{array}$$

where $\zeta_{ij}=\prod_{k=1,k\neq j}^{3}\left(Z_{ij}-Z_{ik}\right)$ and $Z_{ij}\;\left(j=1,2,3\right)$ are roots of equation: $z^{3}+\tau^{-0.5}z^{2}+\tau^{-0.5}\kappa \lambda_{i}^{2}=0$. For γ=−0.5, the sum in Equation (52) should be complemented with a term $\frac{\tau^{\alpha }}{\kappa \lambda_{i}^{2}\sqrt{\pi }}$.

## 4. Numerical Analysis and Discussion

The temperature distribution in the medium is given by the formula which contains the inverse Laplace transform (Equation (50)). This inverse Laplace transform for established values of *α*, *β*, and *γ* can be determined numerically. In the literature, many different algorithms are available for numerical Laplace inversion [29,30,31]. In order to find an effective algorithm for precise numerical inversion of the Laplace transforms appearing in the presented solution, several algorithms were tested. On the basis of these numerical tests, the Fixed-Talbot algorithm for further computations was chosen. Applying this algorithm, the values of a function $f\left(t\right)=L^{-1}\left[\bar{f}\left(s\right)\right]$ are computed using the formula [29]

(53) f(t,M)≈pM{12f¯(p)exp(pt)+∑k=1M−1Re[exp(tμ(θk))f¯(μ(θk))(1+iσ(θk))]}

where μ(θ)=pθ(ctgθ+i), σ(θ)=θ+(θctgθ−1)ctgθ, p=2M/(5t), $\theta_{k}=k\pi /M$, $i=\sqrt{-1}$ and M is the number of precision decimal digits.

Numerical results computed using the Fixed-Talbot procedure were compared with those obtained by using of the analytical form of the inverse Laplace transform (Equation (52)) for the function $U_{1}^{\alpha ,\beta ,\gamma }\left(t\right)$ with α=0.5, β=1.0 and γ=−0.5; 0.0; 0.5. In Table 3, absolute values of the relative errors $E^{\alpha ,\beta ,\gamma }\left(t,k\right)=\left|\frac{{\tilde{U}}_{1}^{\alpha ,\beta ,\gamma }\left(t\right)-U_{1}^{\alpha ,\beta ,\gamma }\left(t\right)}{U_{1}^{\alpha ,\beta ,\gamma }\left(t\right)}\right|$ are presented where ${\tilde{U}}_{1}^{\alpha ,\beta ,\gamma }\left(t\right)$ are obtained by using the numerical inversion of the Laplace transform and $U_{1}^{\alpha ,\beta ,\gamma }\left(t\right)$ are values of the function in Equation (52). The small relative errors justify the use of the Fixed-Talbot procedure for numerical inversion of the Laplace transform given by Equation (50).

The temperature T(x,t) for fixed time *t* as a function of the space variable *x*, defined on the interval [a,b], can achieve a maximum value in the open interval (a,b). The maximum temperature location moves in the medium with a finite speed in the direction appointed by the decreasing temperature gradient. The numerical analysis presented in this section concerns the problem of propagation of the maximum temperature in a finite hollow cylinder that is heated by a heat source or through an operation of variable ambient temperature. Numerical calculations were performed to obtain the following data: The inner and outer radii of the cylinder are a=0.4 m and b=0.6 m, respectively, the thermal diffusivity is $\kappa =8.418\times 10^{-5}\;m^{2}/s$, the thermal conductivity is k=204 W/(m·K), the heat transfer coefficient at the inner surface of the cylinder is $h_{a}=800\;W/\left(m^{2}K\right)$, and the ambient temperature at the initial time *t* = 0 is $T_{a}\left(0\right)=100\;{}^{\circ}C$. In obtaining the numerical results, the following non-dimensional quantities were used: $\hat{x}=\frac{x-a}{b-a}$, $\hat{t}=\frac{\kappa t}{{\left(b-a\right)}^{2}}$, $\hat{\tau }=\frac{\kappa \tau }{{\left(b-a\right)}^{2}}$, $\hat{T}=\frac{T}{T_{a}\left(0\right)}$. The computations were carried out using the Mathematica package [32].

**Figure 1:**
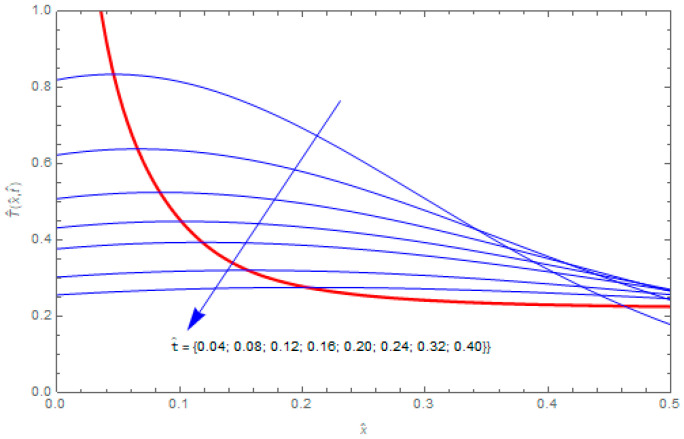
Temperature distributions $\hat{T}\left(\hat{x},\hat{t}\right)$ in the hollow cylinder as functions of the space variable $\hat{x}$ for α=0.15, β=0.9 and different dimensionless time $\hat{t}$.

Let us consider the hollow cylinder heated at the inner surface by the heat source described by the Robotnov function specified by Equation (40) with $g_{0}=10^{8}\;W\cdot s/m$. The graphs of the temperature distributions $\hat{T}\left(\hat{x},\hat{t}\right)$ as functions of the space variable $\hat{x}$ for different moments of time $\hat{t}$ are shown in Figure 1. For a fixed dimensionless time $\hat{t}$, the function of variable $\hat{x}$ assumes a maximum ${\hat{T}}_{\max}$ at a point ${\hat{x}}_{\max}$. The thick red line in Figure 1 is created by the points $\left({\hat{x}}_{\max},{\hat{T}}_{\max}\right)$ which are observed at different times $\hat{t}$. The points of the maximum temperatures propagate with time in the direction of the region of lower temperature. The temperature of the cylinder decreases with time, therefore the maxima, after some time, are small. For this reason, the observation of the temperature maxima is limited to the interval of non-dimensional space variable $\hat{x}\in \left[0,\;0.5\right]$. The presented curves were obtained for α=0.15, β=0.9, $\hat{\tau }=0.01$, and $P_{2}=0$.

Assuming that an operation of the Robotnov heat source defined by Equation (40), the non-dimensional temperature $\hat{T}\left(\hat{x},\hat{t}\right)$ for a fixed value $\hat{t}$ takes a maximal value at $\hat{x}\in \left(0,1\right)$ if the following condition is fulfilled:

(54) $$\frac{\partial \hat{T}\left(\hat{x},\hat{t}\right)}{\partial \hat{x}}=0$$

**Figure 2:**
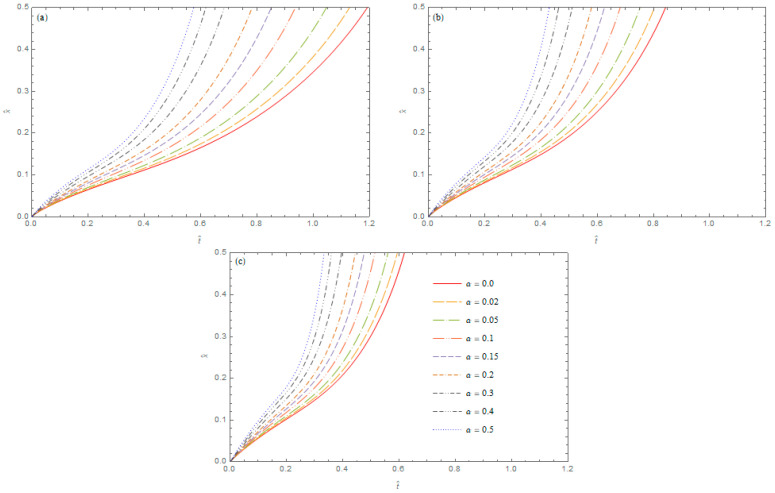
The curves of locations of maxima temperatures in the hollow cylinder for different values of parameter α; (**a**) β=0.9; (**b**) β=0.95; (**c**) β=1.0.

Solving this equation with respect to $\hat{x}$ for $\hat{t}>0$, we obtain a curve of locations of the maxima temperatures in the plane $O\hat{t}\hat{x}$. These curves, for different orders of derivatives *α* and *β*, are presented in Figure 2. The results indicate an important significance of the orders of fractional derivatives occurring in the heat conduction equation for the time of the propagation of the maximal temperature in the cylinder. The time of the propagation of the maximal temperature is significantly 

Shorter for higher values of the derivative orders in the heat conduction model. The curves presented in Figure 2 show that the replacement of the Caputo derivative order β=0.9 by β=1.0 leads to a shortening by half of the transition time of the maximum temperature in the cylinder from $\hat{x}=0$ to $\hat{x}=0.5$, i.e., the change of the derivative order β results in a change of speed of the propagation of the maximum temperature in the medium.

**Figure 3:**
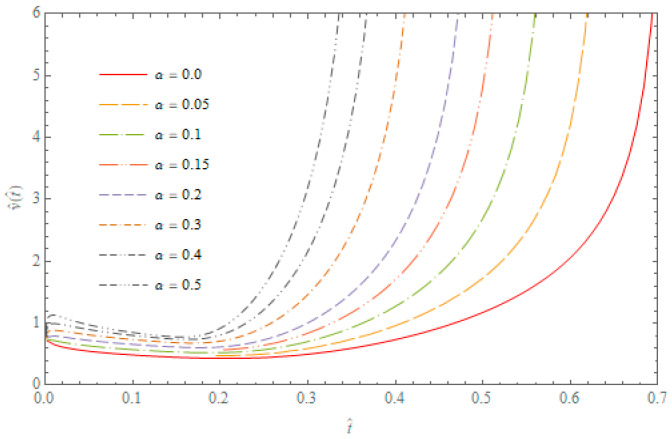
The curves of the propagation speed of maxima temperatures in the hollow cylinder for β=1.0 and different values of parameter α.

The non-dimensional speed of propagation of the maximum temperature $\hat{v}$ is given by the formula v(*t*) = *dx*(*t*)/*dt*, where $\hat{x}\left(\hat{t}\right)$ is an implicit function defined by Equation (54). Differentiating both sides of Equation (54) with respect to $\hat{t}$ and using Equation (16), we find the derivative of the function $\hat{x}\left(\hat{t}\right)$ in the form

(55) $$\hat{v}\left(t\right)=-{\frac{\partial^{2}\hat{\theta }\left(\xi ,\hat{t}\right)}{\partial \xi \partial \hat{t}}/\frac{\partial^{2}\hat{\theta }\left(\xi ,\hat{t}\right)}{\partial \xi^{2}}|}_{\xi =\hat{x}\left(\hat{t}\right)}$$

whereas $\hat{\theta }=\frac{\theta }{T_{a}\left(0\right)}$, ${\frac{\partial \hat{\theta }\left(\xi ,\hat{t}\right)}{\partial \xi }|}_{\xi =\hat{x}\left(\hat{t}\right)}=0$ and ${\frac{\partial^{2}\hat{\theta }\left(\xi ,\hat{t}\right)}{\partial \xi^{2}}|}_{\xi =\hat{x}\left(\hat{t}\right)}\neq 0$. The curves of propagation speed of the maximum temperature in the hollow cylinder subjected to the Robotnov heat source for β=1.0 and different *α* values, are presented in Figure 3. It can be noticed that the propagation speed of the maximum temperature strongly depends on the derivative order *α*, occurring in the fractional heat conduction equation.

**Figure 4:**
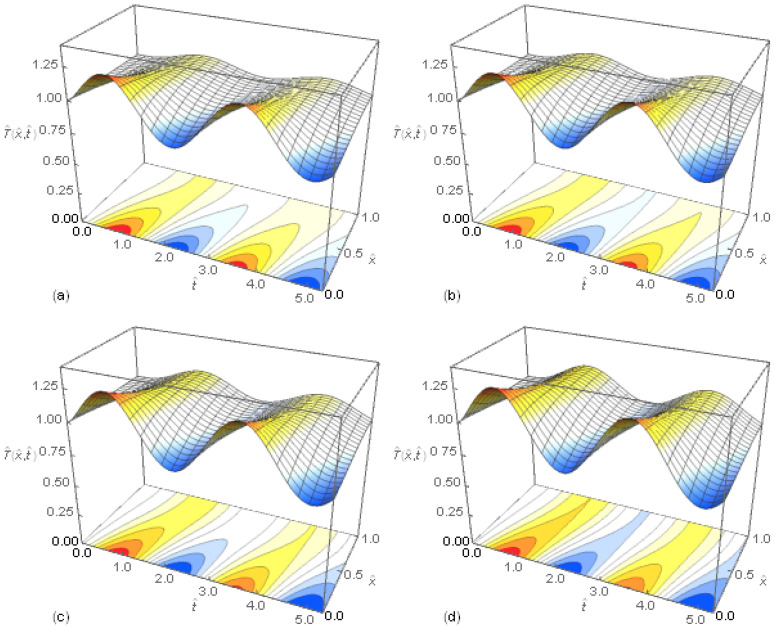
Temperature distributions $\hat{T}\left(\hat{x},\hat{t}\right)$ in the hollow cylinder as functions of non-dimensional space variable $\hat{x}$ and time $\hat{t}$ when the temperature inside the hollow cylinder changes sinusoidaly for the fractional derivative order β=0.9; (**a**) α=0; (**b**) α=0.05; (**c**) α=0.2; (**d**) α=0.5.

The solution presented in the previous section includes a case of the fractional heat conduction in a hollow cylinder when the temperature inside the cylinder changes harmonically according to Equation (46a). The numerical computation of the temperature distribution in the hollow cylinder was performed assuming that no other heat sources occur. In Figure 4, the 3D graphs and contour plots of the function $\hat{T}\left(\hat{x},\hat{t}\right)$ for $P_{2}/P_{1}=0.5$, $\omega =0.005s^{-1}$, β=0.9 and different values of *α* are presented. The maxima and minima of temperatures propagate in the hollow cylinder from the inner to outer boundary. The amplitude of the temperature decreases with the space variable in all cases of the values of *α*. The higher amplitude of the temperature changes at the outer boundary of the cylinder occur for the heat conduction model with the higher order fractional differential equation.

## 5. Conclusions

The solution to the heat conduction problem based on the fractional single-phase-lag model was derived. The formulation and solution of the problem concern the heat conduction in a slab, a hollow cylinder, and a hollow sphere. The considered 1D problem with time-dependent Robin and homogenous Neumann boundary conditions were solved by the use of the eigenfunction expansion method with respect to the space variable and the Laplace transform technique with respect to time. It was assumed that the medium is exposed to a heat source represented by the Robotnov function and the sinusoidaly changing ambient heat. The derived temperature distribution in a hollow cylinder helped in the numerical investigation of propagation of the maximum temperature. It was stated that fractional orders of the Caputo derivatives occurring in the differential equation governing the heat conduction have a significant influence on the propagation of the maximum temperature in the cylinder subjected to the Robotnov heat source. The trajectories of the maximum temperatures show that a slight increase of the fractional derivative orders can cause a considerable decrease of the occurrence time of the maximum temperature in the cylinder. It was observed that the propagation speed of the maximum temperature in the cylinder is higher for a higher fractional order of the differential heat conduction equation. The maximum and minimum temperature propagates in the medium also when the ambient temperature changes harmonically. The amplitude of these changes in the medium decrease with increasing distance from the heated boundary. This decreasing of the amplitudes of the temperature oscillations is greater for smaller orders of the fractional derivative in the heat conduction model. This observation leads to a physical interpretation of the parameter α as a thermal damping coefficient in the fractional heat conduction model.

## Figures and Tables

**Table 1:** The functions $\varphi_{i}\left(x\right)$ and $\psi_{i}\left(x\right)$ for the slab (p=0), hollow cylinder (p=1) and hollow sphere (p=2).

p	*φ_i_* (*x*)	*ψ_i_* (*x*)
0	$\cos\left(\lambda_{i}x\right)$	$\sin\left(\lambda_{i}x\right)$
1	$J_{0}\left(\lambda_{i}x\right)$	$Y_{0}\left(\lambda_{i}x\right)$
2	$\frac{\cos\left(\lambda_{i}x\right)}{x}$	$\frac{\sin\left(\lambda_{i}x\right)}{x}$

**Table 2:** The eigenfunctions $\Phi_{i}\left(x\right)$, eigenvalue equations and normalization integrals $N_{i}$ for the slab (p=0), hollow cylinder (p=1) and hollow sphere (p=2).

p	Eigenfunction	Eigenequation	Normalization Integral
0	$\Phi_{i}\left(x\right)=\cos\left(b-x\right)\lambda_{i}$	$\sin\left(b-a\right)\lambda -\frac{h_{a}}{k\lambda }\cos\left(b-a\right)\lambda =0$	$N_{i}=\frac{b-a}{2}\left(1+\frac{\sin2\lambda_{i}\left(b-a\right)}{2\lambda_{i}\left(b-a\right)}\right)$
1	$\begin{array}{ll} \Phi_{i}\left(x\right) & =Y_{1}\left(b\lambda_{i}\right)J_{0}\left(\lambda_{i}x\right)\\ & -J_{1}\left(b\lambda_{i}\right)Y_{0}\left(\lambda_{i}x\right) \end{array}$	$\begin{array}{l} \frac{h_{a}}{k\lambda }\left(J_{1}\left(b\lambda \right)Y_{0}\left(a\lambda \right)-J_{0}\left(a\lambda \right)Y_{1}\left(b\lambda \right)\right)\\ +J_{1}\left(b\lambda \right)Y_{1}\left(a\lambda \right)-J_{1}\left(a\lambda \right)Y_{1}\left(b\lambda \right)=0 \end{array}$	$\begin{array}{l} \;N_{i}=\frac{2}{\pi^{2}\lambda_{i}^{2}}-\frac{a^{2}}{2}\left(1+{\left(\frac{h_{a}}{k\lambda_{i}}\right)}^{2}\right)\cdot \\ {\left(J_{1}\left(b\lambda_{i}\right)Y_{0}\left(a\lambda_{i}\right)-J_{0}\left(a\lambda_{i}\right)Y_{1}\left(b\lambda_{i}\right)\right)}^{2} \end{array}$
2	$\begin{array}{ll} \Phi_{i}\left(x\right) & =\frac{1}{x}(\cos\lambda_{i}\left(b-x\right)\\ & -\frac{1}{b\lambda_{i}}\sin\lambda_{i}\left(b-x\right)) \end{array}$	$\begin{array}{l} \left(1+\frac{k}{h_{a}}\left(\frac{1}{a}+\frac{1}{b}{\left(b\lambda \right)}^{2}\right)\right)\frac{\sin\left(b-a\right)\lambda }{b\lambda }\\ -\left(1+\frac{k}{h_{a}}\left(\frac{1}{a}-\frac{1}{b}\right)\right)\cos\left(b-a\right)\lambda =0 \end{array}$	$\begin{array}{l} N_{i}=\frac{b^{2}\lambda_{i}^{2}-1}{4b^{2}\lambda_{i}^{3}}\sin2\lambda_{i}\left(b-a\right)+\frac{1}{2b^{2}\lambda_{i}^{2}}\cdot \\ \left(\left(b-a\right){\left(b\lambda_{i}\right)}^{2}-a+b\cos2\lambda_{i}\left(b-a\right)\right) \end{array}$

**Table 3:** The relative errors $E^{\alpha ,\beta ,\gamma }\left(t,k\right)$ of the results obtained by using the Fixed-Talbot procedure and exact values of the function $U_{1}^{\alpha ,\beta ,\gamma }\left(t\right)$ for α=0.5, β=1.0 and γ=−0.5; 0; 0.5.

$\hat{t}=\kappa t/{\left(b-a\right)}^{2}$	γ=−0.5	γ=0	γ=0.5
0.5	$3.36748\times 10^{-7}$	$2.22232\times 10^{-6}$	$2.52736\times 10^{-5}$
1	$4.85927\times 10^{-7}$	$2.84182\times 10^{-6}$	$5.47403\times 10^{-5}$
1.5	$2.51907\times 10^{-7}$	$1.83325\times 10^{-5}$	$5.94386\times 10^{-4}$
2	$5.96577\times 10^{-7}$	$5.66522\times 10^{-6}$	$1.12375\times 10^{-3}$
2.5	$6.85496\times 10^{-7}$	$7.71945\times 10^{-6}$	$1.77174\times 10^{-4}$
